# Prediction of Suicide Attempts and Suicide-Related Events Among Adolescents Seen in Emergency Departments

**DOI:** 10.1001/jamanetworkopen.2022.55986

**Published:** 2023-02-15

**Authors:** David A. Brent, Lisa M. Horowitz, Jacqueline Grupp-Phelan, Jeffrey A. Bridge, Robert Gibbons, Lauren S. Chernick, Margaret Rea, Mary F. Cwik, Rohit P. Shenoi, Joel A. Fein, E. Melinda Mahabee-Gittens, Shilpa J. Patel, Rakesh D. Mistry, Susan Duffy, Marlene D. Melzer-Lange, Alexander Rogers, Daniel M. Cohen, Allison Keller, Robert W. Hickey, Kent Page, T. Charles Casper, Cheryl A. King

**Affiliations:** 1 Department of Psychiatry, University of Pittsburgh School of Medicine, Pittsburgh, Pennsylvania; 2 UPMC Western Psychiatric Hospital, Pittsburgh, Pennsylvania; 3 Intramural Research Program, National Institute of Mental Health, National Institutes of Health, Bethesda, Maryland; 4 Department of Emergency Medicine, UCSF Benioff Children’s Hospitals, Oakland, California; 5 The Abigail Wexner Research Institute at Nationwide Children’s Hospital, Columbus, Ohio; 6 Department of Pediatrics, The Ohio State University College of Medicine, Columbus; 7 Department of Medicine, The University of Chicago, Chicago, Illinois; 8 Department of Public Health Sciences (Biostatistics), The University of Chicago, Chicago, Illinois; 9 Department of Psychiatry and Behavioral Neuroscience, The University of Chicago, Chicago, Illinois; 10 Department of Comparative Human Development, The University of Chicago, Chicago, Illinois; 11 Division of Pediatric Emergency Medicine, Department of Emergency Medicine, Columbia University Irving Medical Center, New York, New York; 12 Department of Emergency Medicine, UC Davis School of Medicine, Sacramento, California; 13 Department of International Health, Social and Behavioral Interventions, Johns Hopkins Bloomberg School of Public Health, Baltimore, Maryland; 14 Division of Emergency Medicine, Department of Pediatrics, Baylor College of Medicine, Houston, Texas; 15 Center for Violence Prevention, Children’s Hospital of Philadelphia, Perelman School of Medicine at University of Pennsylvania, Philadelphia; 16 Division of Emergency Medicine, Department of Pediatrics, Cincinnati Children’s Hospital Medical Center, University of Cincinnati College of Medicine, Cincinnati, Ohio; 17 Division of Pediatric Emergency Medicine, Children’s National Hospital, Washington, DC; 18 Department of Pediatrics, George Washington University School of Medicine and Health Sciences, Washington, DC; 19 Department of Emergency Medicine, George Washington University School of Medicine and Health Sciences, Washington, DC; 20 Department of Pediatrics, University of Colorado School of Medicine, Aurora; 21 Hasbro Children’s Hospital, Department of Pediatrics, Alpert Medical School at Brown University, Providence, Rhode Island; 22 Department of Pediatrics, Medical College of Wisconsin, Milwaukee; 23 Department of Emergency Medicine, University of Michigan, Ann Arbor; 24 Department of Pediatrics, University of Michigan, Ann Arbor; 25 Division of Emergency Medicine, Nationwide Children’s Hospital, Columbus, Ohio; 26 Department of Pediatric Emergency Medicine, University of Utah and Primary Children’s Hospital, Salt Lake City; 27 Department of Pediatrics, University of Pittsburgh, Pittsburgh, Pennsylvania; 28 Department of Pediatrics, University of Utah, Salt Lake City; 29 Department of Psychiatry, Michigan Medicine, Ann Arbor; 30 Injury Prevention Center, The University of Michigan, Ann Arbor

## Abstract

**Importance:**

Screening adolescents in emergency departments (EDs) for suicidal risk is a recommended strategy for suicide prevention. Comparing screening measures on predictive validity could guide ED clinicians in choosing a screening tool.

**Objective:**

To compare the Ask Suicide-Screening Questions (ASQ) instrument with the Computerized Adaptive Screen for Suicidal Youth (CASSY) instrument for the prediction of suicidal behavior among adolescents seen in EDs, across demographic and clinical strata.

**Design, Setting, and Participants:**

The Emergency Department Study for Teens at Risk for Suicide is a prospective, random-series, multicenter cohort study that recruited adolescents, oversampled for those with psychiatric symptoms, who presented to the ED from July 24, 2017, through October 29, 2018, with a 3-month follow-up to assess the occurrence of suicidal behavior. The study included 14 pediatric ED members of the Pediatric Emergency Care Applied Research Network and 1 Indian Health Service ED. Statistical analysis was performed from May 2021 through January 2023.

**Main Outcomes and Measures:**

This study used a prediction model to assess outcomes. The primary outcome was suicide attempt (SA), and the secondary outcome was suicide-related visits to the ED or hospital within 3 months of baseline; both were assessed by an interviewer blinded to baseline information. The ASQ is a 4-item questionnaire that surveys suicidal ideation and lifetime SAs. A positive response or nonresponse on any item indicates suicidal risk. The CASSY is a computerized adaptive screening tool that always includes 3 ASQ items and a mean of 8 additional items. The CASSY’s continuous outcome is the predicted probability of an SA.

**Results:**

Of 6513 adolescents available, 4050 were enrolled, 3965 completed baseline assessments, and 2740 (1705 girls [62.2%]; mean [SD] age at enrollment, 15.0 [1.7] years; 469 Black participants [17.1%], 678 Hispanic participants [24.7%], and 1618 White participants [59.1%]) completed both screenings and follow-ups. The ASQ and the CASSY showed a similar sensitivity (0.951 [95% CI, 0.918-0.984] vs 0.945 [95% CI, 0.910-0.980]), specificity (0.588 [95% CI, 0.569-0.607] vs 0.643 [95% CI, 0.625-0.662]), positive predictive value (0.127 [95% CI, 0.109-0.146] vs 0.144 [95% CI, 0.123-0.165]), and negative predictive value (both 0.995 [95% CI, 0.991-0.998], respectively). Area under the receiver operating characteristic curve findings were similar among patients with physical symptoms (ASQ, 0.88 [95% CI, 0.81-0.95] vs CASSY, 0.94 [95% CI, 0.91-0.96]). Among patients with psychiatric symptoms, the CASSY performed better than the ASQ (0.72 [95% CI, 0.68-0.77] vs 0.57 [95% CI, 0.55-0.59], respectively).

**Conclusions and Relevance:**

This study suggests that both the ASQ and the CASSY are appropriate for universal screening of patients in pediatric EDs. For the small subset of patients with psychiatric symptoms, the CASSY shows greater predictive validity.

## Key Points

**Question:**

How do the Ask Suicide-Screening Questions (ASQ) and the Computerized Adaptive Screen for Suicidal Youth (CASSY) instruments compare in predicting suicide attempts (SAs) among adolescents?

**Findings:**

In a longitudinal cohort study of 2740 youths seen in pediatric emergency departments with a 3-month follow-up, both ASQ and CASSY showed strong predictive validity and similar sensitivity and specificity in predicting future SAs. Both ASQ and CASSY performed similarly among patients with physical symptoms; the CASSY more accurately predicted SAs for those with psychiatric symptoms.

**Meaning:**

This study suggests that for universal screening, both instruments perform well among patients with physical symptoms, but for the small subset of youths with psychiatric symptoms, the CASSY has greater predictive validity.

## Teaser

This cohort study compares the Ask Suicide-Screening Questions instrument with the Computerized Adaptive Screen for Suicidal Youth instrument for the prediction of suicidal behavior among adolescents seen in emergency departments (EDs), across demographic and clinical strata.

## Introduction

The rates of suicidal ideation (SI), suicide attempts (SAs), and suicide have increased among adolescents over the past 15 years, with a sharp increase among Black and Hispanic youths.^1,2,3,4^ There is a growing consensus that screening patients seen in emergency departments (EDs) for suicidal risk is an important component of adolescent suicide prevention.^5^ One-fifth of youths visit an ED at least annually.^6^ Patients presenting to the ED are more likely than the general population to be at risk for suicide; suicide decedents aged 10 to 24 years were nearly 7 times more likely than living controls to have visited an ED within 30 days prior to their death.^7^ A significant proportion of youths seen in pediatric ED settings for physical symptoms have positive screening results for suicide risk.^8,9^ Moreover, there has been an increase in presentations to pediatric EDs for adolescent suicidal behavior that has accelerated since the onset of the COVID-19 pandemic.^1,10,11^ The last clinical contact for a substantial proportion of patients with SAs and for decedents is an ED visit.^7,12,13^ Screening in ED settings may also help to address racial disparities in suicidal behavior and access to care.^2,3,14^

There is less consensus about how to screen for suicide risk. The Emergency Department Study for Teens at Risk for Suicide (ED-STARS) was developed to address this question. ED-STARS, based in the Pediatric Emergency Care Applied Research Network (PECARN), assessed youths for suicidal risk and observed them longitudinally to assess how best to screen for and predict subsequent suicidal behavior. A previous study described the development and validation of the Computerized Adaptive Screen for Suicidal Youth (CASSY).^15^ In this study, we compare the performance of the CASSY with a widely used measure for screening for suicidal risk among patients in EDs, the Ask Suicide-Screening Questions (ASQ).^16^

Although the CASSY was developed explicitly to predict suicidal behavior, the ASQ was originally developed to identify concurrent risk for SI. Nevertheless, both measures show evidence of predictive validity.^13,15,17,18^ The extant findings from studies of these 2 instruments are not easily compared because these studies differed by age range of patients, method and content of outcome assessment, and geographic distribution of EDs.^15,17,18^ The 2 studies demonstrating the predictive validity of the ASQ focused on youths aged 8 to 18 years, using record review of a return to the ED for SI or SA (suicide-related event [SRE]), and were conducted in a single urban ED. The initial validation of the CASSY was based on a network of 13 EDs among adolescents aged 12 to 17 years. The primary outcome for the validation of the CASSY was an SA assessed by follow-up interview.

Herein, we compare the performance of the ASQ and the CASSY with respect to the prediction of our primary outcome of SAs and a secondary outcome of visits to the ED or hospital for an SRE in the 3 months after baseline based on participant and parental report. The performance of the ASQ and the CASSY are compared in subgroups defined by age, sex, race, ethnicity, and presenting chief symptom (physical vs psychiatric). We hypothesized that both instruments would perform significantly better than chance in the prediction of SAs and SREs. In light of racial and ethnic disparities in access to care and in suicidal behavior, we wanted to confirm that both instruments would perform as well for Black and Hispanic youths as for White youths in the prediction of suicidal behavior.^2,3,14,19,20^ The ASQ has demonstrated equivalent psychometric properties for Black and White youths in terms of concurrent suicide risk.^21^ We hypothesized that the CASSY would show greater discriminating value than the ASQ in predicting SAs because of the adaptive and dimensional nature of the CASSY and because the CASSY always administers 3 of the ASQ items.

## Methods

### Study Design and Settings

These data are from ED-STARS, a multicenter, random-series, prospective cohort study supported by PECARN.^22^ Study 1 of ED-STARS is a longitudinal follow-up of 2075 adolescent patients, aged 12 to 17 years, seen in 1 of 13 PECARN EDs from June 26, 2015, through July 31, 2016, and was used to develop the CASSY.^15^ The present study’s evaluation of the ASQ and the CASSY is based on study 2 of ED-STARS, which consists of 2740 adolescents seen in 14 PECARN EDs and 1 Indian Health Service ED between July 24, 2017, and October 29, 2018, who completed a CASSY, an ASQ, and a 3-month follow-up assessment. Derivation of the sample size is provided in eAppendix 1 in Supplement 1. For study 2, the EDs were in the West (4 [26.7%]), Southwest and Central (2 [13.3%]), Midwest (4 [26.7%]), and Mid-Atlantic and New England (5 [33.3%]) regions of the US. By design, 40.3% (1105 of 2740) of the study 2 sample presented with psychiatric chief symptoms. Adolescents completed baseline assessments in the ED on a computer tablet. Interviewers, blinded to baseline data, conducted 3-month computer-assisted telephone follow-up interviews. Adolescent participants received $15 at baseline and $25 or $35 at each follow-up in the form of Amazon.com e-gift cards (hard copy mailed if requested). We obtained institutional review board approval to increase the incentive at follow-up to $35 for nonresponders to obtain more generalizable data. We obtained institutional review board approval from all sites (Morgan Stanley Children’s Hospital of NewYork-Presbyterian, The Children’s Hospital of Philadelphia, Cincinnati Children’s Hospital Medical Center, Children’s National Medical Center, Children’s Hospital of Colorado, Hasbro Children’s Hospital, Children’s Hospital of Wisconsin, C.S. Mott Children’s Hospital–University of Michigan, Nationwide Children’s Hospital, Primary Children’s Hospital, Texas Children’s Hospital, University of Arizona Medical Center, University of California Davis Children’s Hospital, Children’s Hospital of Pittsburgh, and Whiteriver Indian Hospital [Indian Health Service]). Parents or guardians provided written informed consent, and adolescents provided written assent. Parental consent or assessments were conducted in English or Spanish. This study followed the Standards for Reporting of Diagnostic Accuracy (STARD) reporting guideline and used a prediction model to assess outcomes.

### Instruments

#### Ask Suicide-Screening Questions

The ASQ consists of 4 yes-or-no items that assess recent SI, burdensomeness, and lifetime suicidal behavior; a “yes” answer or a nonresponse to any of these items results in a positive screening result for suicide risk.^16,23^ The ASQ showed very high sensitivity (96.9%), specificity (87.6%), and concurrent validity for the identification of clinically significant SI as assessed by a positive score on the Suicidal Ideation Questionnaire (area under the receiver operating characteristic curve [AUROC] = 0.92).^16^ A study of 15 003 youths aged 8 to 18 years, recruited by a combination of universal and targeted screening, showed a sensitivity of 77% and a specificity of 85% for predicting return to the ED for an SRE, as assessed by record review.^17^ A subsequent study in the same ED using universal screening demonstrated a sensitivity of 67%, a specificity of 84%, and an AUROC of 0.75.^18^

#### Computerized Adaptive Screen for Suicidal Youth

The CASSY was developed with data from study 1 and validated with 2754 adolescent ED patients (study 2).^15^ In study 2, adolescents completed the CASSY at baseline, which, as a computerized adaptive test, drew from a pool of 72 items that covered a broad range of risk and protective factors for suicidal behavior. Simulated adaptive testing from the complete response patterns revealed a mean of 11 of the 72 items (mean [SD], 15.3% [5.6%]; range, 5%-21%) per participant. The CASSY always administers 3 ASQ items—past week SI, death wish in past few weeks, and lifetime history of SA—as “anchor items” to ensure coverage of suicidal items in each administration.^24^ Mean (SD) CASSY scores, which are the predicted likelihood of SA within the 3-month follow-up period, were 0.18 (0.11) for those who did make an SA and 0.05 (0.07) for those who did not make an SA. The CASSY had an AUROC of 0.89 in study 1 and 0.87 in study 2 for predicting SAs assessed by 3-month follow-up interviews. Using the 80% specificity threshold from study 1, the CASSY had a sensitivity of 82.4% and specificity of 72.5% (eAppendix 2 in Supplement 1).

Demographic information was obtained from the parent or legal guardian and included self-reported race and ethnicity (American Indian or Alaska Native; Asian, Native Hawaiian or Other Pacific Islander; Black or African American; Hispanic or Latino; White; or multiracial), educational level of parents, and receipt of public assistance. Adolescents also answered 37 to 59 items that characterized the sample on SI and suicidal behavior, nonsuicidal self-injury, depression and anxiety, positive affect, alcohol and drug use, fighting, bullying and victimization, history of concussion, and connectedness to family, friends, and school (eTable 1 in Supplement 1).

### Outcomes

Our primary outcome, an SA between baseline and 3-month follow-up, was defined by (1) an adolescent or parent report of adolescent ED visit or hospitalization with an SA and/or (2) an adolescent responding “yes” to either “In the past 3 months, have you made a suicide attempt?” or “In the past 3 months, have you tried to harm yourself because you were at least partly trying to end your life?” from the adapted Columbia–Suicide Severity Rating Scale.^25^ A secondary outcome, an SRE, was a visit to an ED or hospital for SI or SA based on participant or parental interviews.

### Statistical Analysis

Statistical analysis was performed from May 2021 through January 2023. Descriptive statistics were used to summarize baseline characteristics overall for those with and those without primary (SAs) and secondary (SREs) outcomes by 3 months. Baseline characteristics were compared between those retained or not retained for follow-up. Continuous variables were compared using *t* tests, and categorical variables were compared using χ^2^ tests. Sensitivity, specificity, positive predictive value (PPV), and negative predictive value (NPV) were calculated for prediction of the main and secondary outcomes by the ASQ and by the CASSY at various cut points. The value of the CASSY at which sensitivity plus specificity is maximized was considered the optimal cut point. Sensitivity and specificity of predicting the outcomes by the ASQ and by the CASSY at this optimal cut point were calculated within demographic subgroups and subgroups of adolescents by chief symptoms. The AUROC values for the ASQ and the CASSY were calculated and compared overall and within these same subgroups using the method of DeLong et al.^26^ For these subgroup analyses, α was adjusted using the method of Benjamini and Hochberg^27^ with a false discovery rate set at 5%. The instruments’ specificities were compared between male and female youths and between youths with physical symptoms and youths with psychiatric chief symptoms using χ^2^ tests. The CASSY scores were collected and provided by Adaptive Testing Technologies, which was blinded from outcome and other baseline data.^28^ These scores were provided to the Data Coordinating Center at the University of Utah, where validation analyses were performed. All analyses were conducted using SAS, version 9.4 (SAS Institute Inc).^29^ All *P* values were from 2-sided tests, and results were deemed statistically significant at *P* < .05. Because the CASSY includes 3 of the 4 ASQ items as fixed anchor items, comparison of the AUROC values for the CASSY and the AUROC values for the ASQ essentially tests the improvement in predictive accuracy of the additional adaptively administered items in the CASSY over the 3 ASQ items only.

## Results

### Retention for Follow-up

Of the 6513 eligible adolescents approached for participation, 4050 (62.2%) were enrolled. Adolescents with complete baseline evaluations (3965 [97.9%]) were eligible for follow-up (eTable 2 in Supplement 1; Figure). Among 3933 youths who had completed both baseline CASSY and ASQ instruments, 3-month follow-ups were obtained for 2740 adolescents (69.7% retention; 1705 girls [62.2%] and 991 boys [36.2%]; mean [SD] age at enrollment, 15.0 [1.7] years) from adolescents and parents (2434 [88.8%]), adolescents only (120 [4.4%]), or parents only (186 [6.8%]) (Table 1). The study population included 105 American Indian or Alaska Native adolescents (3.8%); 62 Asian, Hawaiian, or Other Pacific Islander adolescents (2.3%); 469 Black or African American adolescents (17.1%); 678 Hispanic adolescents (24.7%); 1618 White adolescents (59.1%); 161 multiracial adolescents (5.9%); and 325 adolescents (11.9%) with unknown race. A total of 1100 of 2690 participants’ families (40.9%) received public assistance, and 793 participants (28.9%) reported a previous SA. Those who were not retained were more likely to be Black, to have parents with a lower educational level, to live in households receiving public assistance, and to have presented with a psychiatric symptom.^15^

**Figure.**
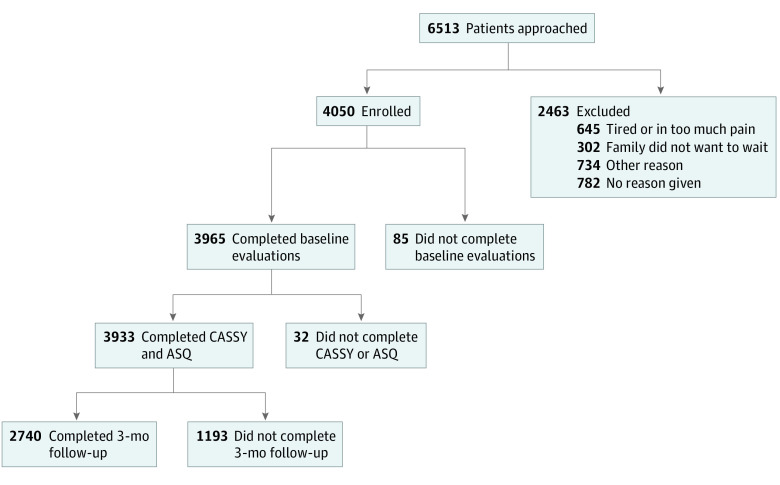
Flow Diagram of Study Participation ASQ indicates Ask Suicide-Screening Questions; CASSY, Computerized Adaptive Screen for Suicidal Youth.

**Table 1.** Baseline Characteristics of Participants Who Made a Suicide Attempt or Visited the ED or Hospital for a Suicide-Related Reason

Characteristic	Participants, No. (%)				
	Yes or no response (N = 2740)	Subjects with 3-mo suicide attempt		Return visit to ED or hospital for suicide attempt or ideation at 3-mo follow-up	
		Yes (n = 163)	No (n = 2577)	Yes (n = 166)	No (n = 2574)
Age at enrollment, mean (SD), y	15.0 (1.7)	14.9 (1.5)	15.0 (1.7)	15.0 (1.6)	15.0 (1.7)
Sex					
Male	991 (36.2)	29 (17.8)	962 (37.3)	37 (22.3)	954 (37.1)
Female	1705 (62.2)	132 (81.0)	1573 (61.0)	126 (75.9)	1579 (61.3)
Unknown	44 (1.6)	2 (1.2)	42 (1.6)	3 (1.8)	41 (1.6)
Race					
American Indian or Alaska Native	105 (3.8)	5 (3.1)	100 (3.9)	0	105 (4.1)
Asian, Native Hawaiian or Other Pacific Islander	62 (2.3)	3 (1.8)	59 (2.3)	4 (2.4)	58 (2.3)
Black or African American	469 (17.1)	33 (20.2)	436 (16.9)	27 (16.3)	442 (17.2)
White	1618 (59.1)	99 (60.7)	1519 (58.9)	110 (66.3)	1508 (58.6)
Multiracial	161 (5.9)	12 (7.4)	149 (5.8)	18 (10.8)	143 (5.6)
Unknown or unavailable	325 (11.9)	11 (6.7)	314 (12.2)	7 (4.2)	318 (12.4)
Ethnicity					
Hispanic or Latino	678 (24.7)	29 (17.8)	649 (25.2)	31 (18.7)	647 (25.1)
Not Hispanic or Latino	1846 (67.4)	124 (76.1)	1722 (66.8)	126 (75.9)	1720 (66.8)
Unknown	216 (7.9)	10 (6.1)	206 (8.0)	9 (5.4)	207 (8.0)
Psychiatric chief symptom	1105 (40.3)	149 (91)	956 (37.1)	155 (93.4)	950 (36.9)
ASQ1: In the past few weeks, have you wished you were dead?	947 (34.6)	143 (87.7)	804 (31.2)	142 (85.5)	805 (31.3)
ASQ2: In the past few weeks, have you felt that you or your family would be better off if you were dead?	831 (30.3)	127 (77.9)	704 (27.3)	121 (72.9)	710 (27.6)
ASQ3: In the past week, have you been having thoughts about killing yourself?	837 (30.5)	133 (81.6)	704 (27.3)	133 (80.1)	704 (27.4)
ASQ4: Have you ever tried to kill yourself?	705 (25.7)	123 (75.5)	582 (22.6)	107 (64.5)	598 (23.2)
Suicide attempt–lifetime^a^	793 (28.9)	133 (81.6)	660 (25.6)	116 (69.9)	677 (26.3)
CASSY: probability of a future suicide attempt, median (IQR)	0.03 (0.00-0.09)	0.15 (0.10-0.24)	0.02 (0.00-0.08)	0.14 (0.09-0.21)	0.02 (0.00-0.08)
ASQ: positive answer to any ASQ item	1217 (44.4)	155 (95.1)	1062 (41.2)	153 (92.2)	1064 (41.3)

Abbreviations: ASQ, Ask Suicide-Screening Questions; CASSY, Computerized Adaptive Screen for Suicidal Youth; ED, emergency department.

^a^ Patients who reported having ever tried to kill themselves, made a suicide attempt, or tried to harm themselves because they were at least partly trying to end their life.

### SAs and SREs

Of the 2740 participants with 3-month follow-up, 234 (8.5%) had an SA, an SRE, or both. Specifically, 163 (5.9%) had at least 1 SA, 166 (6.1%) had an SRE, 95 (3.5%) had both an SA and an SRE, 68 (2.5%) had an SA only, and 71 (2.6%) reported an SRE only (Table 1).

### ASQ and CASSY Performance

There were no significant differences between the ASQ and the CASSY with respect to sensitivity (0.951 [95% CI, 0.918-0.984] vs 0.945 [95% CI, 0.910-0.980]), specificity (0.588 [95% CI, 0.569-0.607] vs 0.643 [95% CI, 0.625-0.662]), PPV (0.127 [95% CI, 0.109-0.146] vs 0.144 [95% CI, 0.123-0.165]), or NPV (both 0.995 [95% CI, 0.991-0.998]) in predicting an SA within 3 months, respectively, with similar findings for prediction of an SRE. Table 2 and eTable 3 in Supplement 1 show the comparison of the ASQ and the CASSY when either the specificity or the sensitivity of the CASSY is set to that of the ASQ for SAs and SREs, respectively. Across demographic and clinical stratifications, there were no differences between the CASSY and the ASQ with respect to sensitivity, specificity, PPV, or NPV.

**Table 2.** Prediction of 3-Month Suicide Attempt

Screening questionnaire	Sensitivity (95% CI)	Specificity (95% CI)	Positive predictive value (95% CI)	Negative predictive value (95% CI)
ASQ	0.951 (0.918-0.984)	0.588 (0.569-0.607)	0.127 (0.109-0.146)	0.995 (0.991-0.998)
CASSY (using cut point of 0.0447, where sensitivity is equal to sensitivity for ASQ)	0.951 (0.918-0.984)	0.622 (0.603-0.640)	0.137 (0.117-0.157)	0.995 (0.992-0.998)
CASSY (using cut point of 0.0408, where specificity is equal to specificity for ASQ)	0.963 (0.934-0.992)	0.588 (0.569-0.607)	0.129 (0.110-0.148)	0.996 (0.993-0.999)
CASSY (using optimal cut point of 0.0436, where sensitivity plus specificity is maximized)	0.945 (0.910-0.980)	0.643 (0.625-0.662)	0.144 (0.123-0.165)	0.995 (0.991-0.998)

Abbreviations: ASQ, Ask Suicide-Screening Questions; CASSY, Computerized Adaptive Screen for Suicidal Youth.

The CASSY’s AUROC was significantly higher than that of the ASQ for predicting both SAs (0.867 [95% CI, 0.845-0.888] vs 0.769 [95% CI, 0.750-0.789]; *P* < .001) (Table 3) and SREs (0.841 [95% CI, 0.818-0.864] vs 0.754 [95% CI, 0.732-0.777]; *P* < .001) (eTable 4 in Supplement 1). The AUROCs for both instruments were significantly higher than that obtained by predicting future SAs based on age, sex, race, and ethnicity (AUROC, 0.55 [95% CI, 0.51-0.60]). The AUROC for using ASQ item 3 only (past-week SI) was similar to the AUROC obtained using the 4-item ASQ (AUROC, 0.77 [95% CI, 0.74-0.80]).

**Table 3.** AUROC, Sensitivity, and Specificity for Predicting 3-Month Suicide Attempt by Screening Questionnaire Within Subgroups

Subgroup	No.	AUROC (95% CI)			Sensitivity (95% CI)		Specificity (95% CI)	
		CASSY	ASQ	*P* value^a^	CASSY^b^	ASQ	CASSY^b^	ASQ
Overall	2740	0.867 (0.845-0.888)	0.769 (0.750-0.789)	<.001	0.945 (0.910-0.980)	0.951 (0.918-0.984)	0.643 (0.625-0.662)	0.588 (0.569-0.607)
Sex								
Male	991	0.880 (0.831-0.929)	0.793 (0.735-0.852)	<.001	0.862 (0.737-0.988)	0.897 (0.786-1.000)	0.758 (0.731-0.785)	0.690 (0.661-0.719)
Female	1705	0.844 (0.817-0.871)	0.743 (0.723-0.764)	<.001	0.962 (0.930-0.995)	0.962 (0.930-0.995)	0.571 (0.546-0.595)	0.524 (0.500-0.549)
Age, y								
12-14	1339	0.870 (0.841-0.900)	0.780 (0.752-0.808)	<.001	0.931 (0.878-0.984)	0.943 (0.894-0.991)	0.669 (0.642-0.695)	0.617 (0.590-0.644)
15-17	1401	0.867 (0.836-0.898)	0.760 (0.734-0.786)	<.001	0.961 (0.917-1.000)	0.961 (0.917-1.000)	0.620 (0.593-0.646)	0.560 (0.533-0.587)
Race								
Black or African American	469	0.847 (0.791-0.903)	0.756 (0.701-0.811)	<.001	0.879 (0.767-0.990)	0.909 (0.811-1.000)	0.679 (0.635-0.723)	0.603 (0.557-0.649)
White	1618	0.864 (0.836-0.891)	0.773 (0.752-0.794)	<.001	0.970 (0.936-1.000)	0.970 (0.936-1.000)	0.614 (0.590-0.639)	0.577 (0.552-0.602)
Other or unknown	653	0.895 (0.851-0.938)	0.770 (0.722-0.818)	<.001	0.935 (0.849-1.000)	0.935 (0.849-1.000)	0.690 (0.653-0.726)	0.605 (0.566-0.643)
Ethnicity								
Hispanic	678	0.856 (0.809-0.903)	0.787 (0.748-0.826)	.003	0.897 (0.786-1.000)	0.966 (0.899-1.000)	0.683 (0.647-0.718)	0.609 (0.571-0.646)
Not Hispanic	1846	0.867 (0.841-0.893)	0.759 (0.736-0.783)	<.001	0.952 (0.914-0.989)	0.944 (0.903-0.984)	0.624 (0.601-0.647)	0.575 (0.552-0.598)
Psychiatric chief symptom								
No	1635	0.938 (0.914-0.961)	0.878 (0.807-0.949)	.07	1.000 (1.000-1.000)	0.929 (0.794-1.000)	0.877 (0.861-0.893)	0.827 (0.809-0.846)
Yes	1105	0.724 (0.681-0.767)	0.568 (0.547-0.588)	<.001	0.940 (0.901-0.978)	0.953 (0.919-0.987)	0.248 (0.221-0.275)	0.182 (0.158-0.206)

Abbreviations: ASQ, Ask Suicide-Screening Questions; AUROC, area under the receiver operating characteristic curve; CASSY, Computerized Adaptive Screen for Suicidal Youth.

^a^ All *P* values significant after using the Benjamini-Hochberg procedure except the *P* value of .07 for patients with physical chief symptoms.

^b^ Using optimal cut point.

For predicting SAs, the CASSY showed higher AUROCs than the ASQ for demographic stratifications by age, sex, race, and ethnicity (Table 3). For patients presenting with physical chief symptoms, the AUROCs for the CASSY and the ASQ were both high and not significantly different (0.938 [95% CI, 0.914-0.961] vs 0.878 [95% CI, 0.807-0.949], respectively; *P* = .07); for those with psychiatric chief symptoms, the CASSY had a higher AUROC than the ASQ (0.724 [95% CI, 0.681-0.767] vs 0.568 [95% CI, 0.547-0.588], respectively; *P* < .001). For both measures, the specificity was lower in female youths than male youths (CASSY, 0.571 [95% CI, 0.546-0.595] vs 0.758 [95% CI, 0.731-0.785], respectively; ASQ, 0.524 [95% CI, 0.500-0.549] vs 0.690 [95% CI, 0.661-0.719], respectively; *P* < .001 for both), and for those presenting with psychiatric vs physical symptoms (CASSY, 0.248 [95% CI, 0.221-0.275] vs 0.877 [95% CI, 0.861-0.893], respectively; ASQ, 0.182 [95% CI, 0.158-0.206] vs 0.827 [95% CI, 0.809-0.846], respectively; *P* < .001 for both). In comparisons within each measure, the AUROCs were similar among Black, Hispanic, and White youths.

The median CASSY scores were 0.00 (range, 0.00-0.03) for those who presented with physical symptoms and 0.10 (range, 0.05-0.16) for those who presented with psychiatric symptoms. The individual ASQ items are presented for comparison in Table 4.

**Table 4.** CASSY and ASQ Questions by Subgroup^a^

Subgroup	No.	CASSY, median (IQR)	Participants, No. (%)			
			ASQ1	ASQ2	ASQ3	ASQ4
Sex						
Male	991	0.01 (0.00-0.05)	230 (23.2)	187 (18.9)	206 (20.8)	167 (16.9)
Female	1705	0.04 (0.00-0.12)	708 (41.5)	633 (37.1)	624 (36.6)	528 (31.0)
Unknown	44	0.03 (0.00-0.06)	9 (20.5)	11 (25.0)	7 (15.9)	10 (22.7)
Age group, y						
12-14	1339	0.02 (0.00-0.08)	441 (32.9)	386 (28.8)	395 (29.5)	309 (23.1)
15-17	1401	0.03 (0.00-0.10)	506 (36.1)	445 (31.8)	442 (31.5)	396 (28.3)
Race						
Black or African American	469	0.02 (0.00-0.08)	147 (31.3)	140 (29.9)	126 (26.9)	122 (26.0)
White	1618	0.03 (0.00-0.10)	607 (37.5)	528 (32.6)	540 (33.4)	431 (26.6)
Other or unknown	653	0.02 (0.00-0.07)	193 (29.6)	163 (25.0)	171 (26.2)	152 (23.3)
Ethnicity						
Hispanic	678	0.02 (0.00-0.07)	206 (30.4)	191 (28.2)	180 (26.5)	174 (25.7)
Not Hispanic	1846	0.03 (0.00-0.10)	672 (36.4)	582 (31.5)	598 (32.4)	481 (26.1)
Unknown	216	0.02 (0.00-0.08)	69 (31.9)	58 (26.9)	59 (27.3)	50 (23.1)
Psychiatric chief symptom						
No	1635	0.00 (0.00-0.03)	137 (8.4)	140 (8.6)	74 (4.5)	148 (9.1)
Yes	1105	0.10 (0.05-0.16)	810 (73.3)	691 (62.5)	763 (69.0)	557 (50.4)

Abbreviations: ASQ, Ask Suicide-Screening Questions; CASSY, Computerized Adaptive Screen for Suicidal Youth.

^a^ Column definitions: CASSY, probability of a future suicide attempt; ASQ1: In the past few weeks, have you wished you were dead?; ASQ2: In the past few weeks, have you felt that you or your family would be better off if you were dead?; ASQ3: In the past week, have you been having thoughts about killing yourself?; and ASQ4: Have you ever tried to kill yourself?

## Discussion

In this prospective, multicenter cohort study of adolescent patients seen in the ED, we compared the performance of the ASQ, a 4-item screening tool for suicide risk, with the CASSY, a computerized adaptive test that always includes 3 items from the ASQ and, on average, 8 additional items. Among patients with physical symptoms, accounting for most ED presentations, the AUROCs for predicting SAs in both measures were high and indistinguishable. There were no differences between the CASSY and the ASQ with respect to sensitivity, specificity, PPV, or NPV overall or for any stratification for either primary or secondary outcomes. However, the CASSY had a higher AUROC overall, across several demographic strata, and among those who presented with psychiatric reasons for the prediction of SAs and SREs.

The main advantages of the ASQ are that it is brief (4 items), free of charge, has widespread use and validation in multiple settings and age groups, and is integrated into youth suicide risk clinical pathways.^30,31,32,33,34,35,36,37^ In a sample in which most youths present with physical symptoms, the performance of the ASQ will be indistinguishable from the CASSY. Although the ASQ was initially validated against a concurrent measure of SI, its ability to predict future SAs is logical given the association between more severe SI and future suicidal behavior.^38^ Although the AUROCs for the prediction of SAs were similar for the ASQ item 3 and the 4-item ASQ, we caution against a single-item measure in light of findings that SI is not as strong a predictor of future SAs for Black youths compared with White youths.^19^ In addition, predicting an SA is not the only outcome of interest, given that youths with SI are highly likely to need a mental health referral.^38^

The primary advantage of the CASSY is that it is a dimensional measure that provides both a continuous severity score and an estimate of the probability of an SA in the next 3 months. This continuous severity score can be used to track changes in suicidal risk over time. Clinical pathways can be developed based on stratifications of the estimated probability of an SA. Finally, the CASSY allows end users flexibility to make trade-offs between sensitivity and specificity.^39^ The CASSY shows stronger predictive validity in patients with psychiatric chief complaints.

The main disadvantages of the ASQ are that it is a dichotomous measure and that its predictive validity for those presenting with behavioral health complaints is lower than that of the CASSY. The main disadvantages of the CASSY are that it is not free, it requires a license and a computer interface, and, while brief, it is longer than the ASQ (4 items vs a mean of 11 items). Some sites may be reluctant to document an estimated probability of an SA for medicolegal reasons. Both measures showed lower specificity in predicting SAs for female youths vs male youths and lower specificity in predicting SREs for physical vs psychiatric symptoms.

The ASQ and the CASSY will both function effectively as suicide risk screening tools for youths presenting to EDs. For universal screening, among most patients in the ED, the ASQ and the CASSY both showed equally high sensitivity and specificity. Both instruments show similar performance for Black, Hispanic, and White youths. No cost, simplicity in administration, brevity, and its integration into a care pathway favor the ASQ. Flexibility to customize sensitivity and specificity, a dimensional output that provides the likelihood of predicting an SA, and higher accuracy for predicting SAs for those with behavioral health symptoms are the main advantages of the CASSY. Using these tools as part of a clinical pathway,^30,35^ which may include further assessments with the ASQ Brief Suicide Safety Assessment^23^ or the Columbia Suicide Severity Rating Scale,^25^ may provide efficient triaging for those at greatest risk. Studies conducted among adults suggest that both tools should be paired with brief interventions to promote safety planning and linkage to services that can reduce the risks for a subsequent SA.^40^

### Strengths and Limitations

This study has some strengths, including a sample that is geographically, racially, and ethnically diverse and drawn from 15 different ED settings; parental consent and interviews offered in English or Spanish; moderate acceptance and follow-up rates; and the assessment of outcomes by clinical interview rather than record review.

This study also has some limitations, including recruitment primarily from academic medical centers, as well as greater attrition among Black patients, those presenting with psychiatric symptoms, and those whose parents were poorer and had less education. Although specificity was low in both instruments for predicting future SAs, most individuals with a positive screening test for suicidal risk will have at least one lifetime psychiatric disorder, so a mental health referral is often indicated.^38^ Future studies can examine clinician and patient preference with respect to instrument features and performance as part of a clinical pathway designed to engage patients with SI in treatment and prevent future SAs.

## Conclusions

In this cohort study, both the CASSY and the ASQ performed well in predicting SAs and SREs among patients presenting with physical symptoms. The CASSY performed better than the ASQ in predicting future SAs among patients with psychiatric concerns, who constitute a relatively small but consequential and growing proportion of pediatric ED attendees. The similarity of the 2 measures in sensitivity, specificity, PPV, and NPV suggest that both instruments will perform well for universal screening in EDs. Emergency department clinicians will need to decide which measure is best suited to their milieu and patient mix. Both the ASQ and the CASSY are worthy of consideration for identifying youths in the ED at risk for suicide.

## Notes

Supplement 1. **eAppendix 1.** Derivation of Sample Size **eAppendix 2.** Additional Information on the CASSY **eTable 1.** List of Measures Used for Baseline and Follow-up Assessments **eTable 2.** Characteristics of Adolescents Retained and Lost to Follow-up **eTable 3.** Predicting Return Visit to ED/Hospital for Suicide Attempt/Ideation at 3-Month Follow-up (Measure and 95% CI) **eTable 4.** AUC, Sensitivity, and Specificity for Predicting Return Visit to ED/Hospital for Suicide Attempt/Ideation at 3-Month Follow-up by Screening Questionnaire Within Subgroups (Measure and 95% CI)
eReferences.

Supplement 2. **Nonauthor Collaborators.** Pediatric Emergency Care Applied Research Network (PECARN) Collaborators

Supplement 3.
Data Sharing Statement
