# Supplementary material for: Prediction of Suicide Attempts and Suicide-Related Events Among Adolescents Seen in Emergency Departments
Source: JAMA Netw Open. 2023 Feb 15;6(2):e2255986. doi: 10.1001/jamanetworkopen.2022.55986 (PMC9932829; doi:10.1001/jamanetworkopen.2022.55986)
Supplement: Supplement 1. — eAppendix 1. Derivation of Sample Size eAppendix 2. Additional Information on the CASSY eTable 1. List of Measures Used for Baseline and Follow-up Assessments eTable 2. Characteristics of Adolescents Retained and Lost to Follow-up eTable 3. Predicting Return Visit to ED/Hospital for Suicide Attempt/Ideation at 3-Month Follow-up (Measure and 95% CI) eTable 4. AUC, Sensitivity, and Specificity for Predicting Return Visit to ED/Hospital for Suicide Attempt/Ideation at 3-Month Follow-up by Screening Questionnaire Within Subgroups (Measure and 95% CI) eReferences. [file jamanetwopen-e2255986-s001.pdf]

Supplementary Online Content

Brent DA, Horowitz LM, Grupp-Phelan J, et al; Pediatric Emergency Care Applied Research Network (PECARN). Prediction of suicide attempts and suicide-related events among adolescents seen in emergency departments. *JAMA Netw Open.* 2023;6(2):e2255986. doi:10.1001/jamanetworkopen.2022.55986

eAppendix 1. Derivation of Sample Size
eAppendix 2. Additional Information on the CASSY
eTable 1. List of Measures Used for Baseline and Follow-up Assessments
eTable 2. Characteristics of Adolescents Retained and Lost to Follow-up
eTable 3. Predicting Return Visit to ED/Hospital for Suicide Attempt/Ideation at 3-Month Follow-up (Measure and 95% CI)
eTable 4. AUC, Sensitivity, and Specificity for Predicting Return Visit to ED/Hospital for Suicide Attempt/Ideation at 3-Month Follow-up by Screening Questionnaire Within Subgroups (Measure and 95% CI)
eReferences.

This supplementary material has been provided by the authors to give readers additional information about their work.

eAppendix 1. Derivation of Sample Size

Based on prior studies, we estimated that sensitivity of the ASQ was approximately 0.80. With the sample size we expected to enroll, of which 40% were those with behavioral health complaints and 60% with medical complaints, and with 80% follow-up, we predicted that approximately 131 youth would report an SA at 3 months. With this number, we calculated that we could detect a difference in sensitivity of about 0.09-0.11 with 80% power. For AUC, past studies had reported values between about 0.6 and 0.7 for SA screening. Based on the proposed sample size, about 100 SAs would be required for 80% power to detect a difference in AUC of 0.09.

Our actual rate of attrition was around 70%, but we ended up with a higher number of suicide attempts than predicted, namely 166, so that we had adequate power to detect our planned difference in AUC.

© 2023 Brent DA et al. *JAMA Network Open.*

eAppendix 2. Additional Information on the CASSY

The ASQ is a fixed-length short form (4-item) questionnaire in which all people are asked four questions regarding suicidal behavior and ideation. Endorsement of any single item leads to a suicide risk warning, which results in a dichotomous classification. The CASSY is a computerized adaptive test based on multidimensional item response theory. It uses information from a bank of 72 items drawn from the domains of suicidal ideation and behavior, psychopathology, PTSD, social adjustment, sleep, anger/aggression, and substance use. With an average of 11 items, a correlation of r=0.94 with the 72-item bank is maintained. Median administration time is 1 minute and 24 seconds. The CASSY adaptively selects an optimal set of items for each individual on each measurement occasion, by targeting the severity level of the items to the suicidality level of the individual, which it learns through the adaptive administration process. Unlike the ASQ which produces a binary risk indicator, the CASSY provides a continuous suicidality severity score ranging from 0-100 with 5 points of precision, as well as an estimate of the probability that the subject will make a suicide attempt in the next 3 months. The risk probability can be dichotomized at different points depending on the balance between sensitivity and specificity desired by the user. For example, fixing specificity at 80% and 90% in Study 1 resulted in sensitivities of 83% and 61%, respectively.

There are several unique advantages of the CASSY:

First, it provides both a continuous suicide severity score and a suicide risk probability that can be used to provide a much finer grained analysis of suicide severity and risk than the simple binary classification.

Related to the first point, the CASSY severity score can be used to measure change in a much more fine-grained manner than a simple binary classification.

Third, if a person does not report suicidal ideation or behavior on the ASQ, they cannot screen positive and are considered not at risk. The same is not true for the CASSY, where items that load on the primary suicidality dimension from other domains such as related psychopathology, PTSD, and social adjustment to convey risk quantified by the CASSY severity score and risk estimate even in the absence of suicidal ideation and behavior.

Fourth, depending on the application, the CASSY severity score and/or risk probability can be dichotomized at different points to alter the balance between sensitivity and specificity. As an example, at high specificity of 0.90, sensitivity is still 0.61 for a suicide attempt in the next 3 months. Of course, some of those estimated to be at risk will make suicide attempts beyond the 3-month window, so this is a lower bound on the true positive rate.

© 2023 Brent DA et al. *JAMA Network Open.*

eTable 1. List of Measures Used for Baseline and Follow-up Assessments

Domain	Measure	# Items	Sample item(s)	Parent or Youth Report	Baseline or Follow-up	Comments
Suicidality	Ask Suicide-Screening Questions (ASQ) [1]	4	ASQ-3: In the past week, have you been having thoughts about killing yourself?	Youth	Baseline	Summarized in Table 1 but not considered as predictor for outcome models
	Columbia Suicide Severity Rating Scale (C-SSRS) [2]	10	Have you ever in your life made a suicide attempt?	Youth	2 items at baseline, 10 at 3, 6 months	Baseline includes lifetime suicide attempt history. Follow-ups cover severity of thoughts, attempts, other suicidal behavior, severity of suicidal ideation since previous assessment
Depression and Generalized Anxiety	Patient Health Questionnaire (PHQ-4) [3,4]	4 (2 items each)		Youth	Baseline, follow-up	First two items of the PHQ-9 which measure mood and anhedonia; first two items of the GAD-7, which measure generalized anxiety. Only baseline responses included in analysis
Alcohol Use	Alcohol Use Disorders Identification Test-Consumption (AUDIT) [5]	3		Youth	Baseline, follow-up	Only baseline included in analysis

eTable 1. List of Measures Used for Baseline and Follow-up Assessments (continued)

Domain	Measure	# Items	Sample item(s)	Parent or Youth Report	Baseline or Follow-up	Comments
Drug Use	National Institute on Drug Abuse - Modified Alcohol, Smoking and Substance Involvement Screening Test (version 31) of the Drug Use Scale [6,7]	4 (and up to 3 additional)		Youth	Baseline, follow-up	Only baseline included in analysis
Fights	Youth Risk Behavior Survey [8,9]	1	Frequency of fights	Youth	Baseline, follow-up	Only baseline responses included in analysis
Externalizing symptoms	Subscale from Pediatric Symptom Checklist [10,11]	7		Parent	Baseline	Sum of items (allowing up to one missing item, regarded as 0)
Inattention	Subscale from Pediatric Symptom Checklist [10,11]	5		Parent	Baseline	Sum of items (allowing up to one missing item, regarded as 0)
Non-suicidal self-injury (NSSI)	Youth Risk Behavior Survey [12]<br><br>Functional Assessment of Self-Mutilation[13]	2	In the past 12 months, have you ever harmed or hurt your body on purpose, such as cutting or burning your skin, or hitting yourself, without wanting to die?	Youth	Baseline, follow-up	If any instances in the past 12 months, the method(s) used were requested and tallied

© 2023 Brent DA et al. *JAMA Network Open.*

eTable 1. List of Measures Used for Baseline and Follow-up Assessments (continued)

Domain	Measure	# Items	Sample item(s)	Parent or Youth Report	Baseline or Follow-up	Comments
Peer victimization	Peer Victimization and Perpetration Questionnaire—Peer Victimization, and Bully Perpetration [14,15,16]	4 (2 items each)		Youth	Baseline	Only the perpetration subscale was included in analysis
Connectedness	Parent-Family Connectedness Scale [17]	2	How much do people in your family understand you?	Youth	Baseline, follow-up	5-level ordinal items. Only baseline responses used for analysis
	School Connectedness Scale [17]	2	You feel like you are a part of your school.	Youth	Baseline, follow-up	5-level Likert items from strongly disagree to strongly agree. Only baseline responses used for analysis
	How I feel about friends from Hemingway's Adolescent Connectedness Scale [18]	2	I have friends I'm really close to and trust completely.	Youth	Baseline, follow-up	5-level ordinal items. Only baseline responses used for analysis
Sexual identity	Sexual Identity Behavior and Attraction Scale [19,20,21]	1	Do you see yourself as… (straight, mostly straight, etc.)?	Youth	Baseline	Used to create a sexual minority variable (minority if and only if anything besides straight was selected

© 2023 Brent DA et al. *JAMA Network Open.*

eTable 1. List of Measures Used for Baseline and Follow-up Assessments (continued)

Domain	Measure	# Items	Sample item(s)	Parent or Youth Report	Baseline or Follow-up	Comments
Positive feelings	Positive Affect Subscale of the shortened Positive and Negative Affect Schedule for children (PANAS-C) [22,23]	5		Youth	Baseline, follow-up	Only baseline responses used for analysis.
Demographics	Age, sex at birth, racial and ethnic identification, parental education levels, welfare status			Parent	Baseline	

The four-item Ask Suicide-Screening Questions (ASQ) scale was administered to assess suicidal ideation and lifetime suicide attempts.[1] Two items from the Columbia Suicide Severity Rating Scale (C-SSRS) were administered to assess lifetime and recent (i.e., past month and past 24-hour) suicide attempts.[2] Youth who reported a suicide attempt responded to two items from the C-SSRS Behavior Scale to indicate the method and approximate date of their most recent attempt.[2]

The four-item PHQ-4 was used to measure depressive symptomology over the past two weeks.[3,4] Youth responded to statements on a 4-point ordinal scale with options ranging from "not at all" to "nearly every day."

The Alcohol Use Disorder Identification Test-Consumption (AUDIT-C) was used to ascertain drinking frequency and quantity.[5] Each item in the 3-item screen is scored on a 4-point scale. A sample item is: "How many drinks with alcohol in it do you have on a day when you are drinking?" Youth completed the adapted four-item Drug Use Scale to assess illicit drug use in the past 3 months.[6,7] Drug categories included cannabis, prescription stimulants, prescribed opioids, and sedatives or sleeping pills, and items were scored using a 5-point ordinal scale ranging from "never" to "daily or almost daily." For 3 of the items, youth who indicated drug use were presented with an additional item to assess drug abuse determined by drug use to get high, use in excess, or using a prescription prescribed to someone else. One item from the Youth Risk Behavior Survey captured frequency of fighting in the past 12 months. The item is as follows: "During the

past 12 months, how many times were you in a physical fight?”[8,9] Externalizing and inattention symptoms were assessed from the corresponding sub-scales of the Pediatric Symptom Checklist.[10,11]

Non-suicidal self-injury (NSSI) frequency was assessed with the Youth Behavior Risk Survey (YRBS)[12] item: “In the past 12 months, have you ever harmed or hurt your body on purpose, such as cutting or burning your skin, or hitting yourself, without wanting to die?” Response options were: 0 times, 1-2 times, 3-4 times, or 5 or more times. An additional item derived from the Functional Assessment of Self-mutilation (FASM)[13] assessed the number of NSSI methods: “Over the last 12 months, which method(s) have you used to hurt yourself?” Sample methods include “scraping skin to draw blood” and “cutting or carving on skin.”

Four items were used from the Peer Victimization and Bullying Perpetration to assess bullying-victimization in school and away from school and bullying perpetration in school and away from school.[14,15,16]

Connectedness was measured with both items from the Parent-Family Connectedness Scale,[17] two items from the How I Feel About Friends Scale,[18] and both items from the How I Feel About School Scale.[17]

The Sexual Identity Behavior and Attraction Scale was also administered at baseline to youth to assess current gender identity, sexual orientation, and lifetime sexual behavior and romantic attraction. Example items include: “What is your current gender identity?” and “During your life, with whom have you had sexual contact (not including unwanted experiences)?”[19,20,21]

The 5-item Positive Affect Subscale queried positive feelings over the past few weeks.[22,23]

© 2023 Brent DA et al. *JAMA Network Open.*

eTable 2. Characteristics of Adolescents Retained and Lost to Follow-up

	Retained (N = 2740)	Lost to Follow-up (N = 1193)	P-value
**Age at enrollment (years)**: Mean (SD)	15.0 (1.65)	15.0 (1.69)	0.219[1]
**Sex**: Male	991/2740 (36%)	437/1193 (37%)	0.943[2]
**Race**			
American Indian or Alaska Native	105/2740 (4%)	15/1193 (1%)	<.001[2]
Asian or Native Hawaiian or Other Pacific Islander	62/2740 (2%)	19/1193 (2%)	
Black or African American	469/2740 (17%)	292/1193 (24%)	
White	1618/2740 (59%)	634/1193 (53%)	
Multi-racial	161/2740 (6%)	72/1193 (6%)	
Unknown or unavailable	325/2740 (12%)	161/1193 (13%)	
**Ethnicity**			
Hispanic	678/2740 (25%)	306/1193 (26%)	0.001[2]
Not Hispanic	1846/2740 (67%)	752/1193 (63%)	
Unknown	216/2740 (8%)	135/1193 (11%)	
**Education - parent 1**			
High school graduate or less	799/2692 (30%)	443/1178 (38%)	<.001[2]
Some college/technical training	607/2692 (23%)	318/1178 (27%)	
College graduate/professional training	1270/2692 (47%)	406/1178 (34%)	
Don't know/Not applicable	16/2692 (1%)	11/1178 (1%)	
**Education - parent 2**			
High school graduate or less	992/2628 (38%)	507/1143 (44%)	<.001[2]
Some college/technical training	445/2628 (17%)	179/1143 (16%)	
College graduate/professional training	908/2628 (35%)	291/1143 (25%)	
Don't know/Not applicable	283/2628 (11%)	166/1143 (15%)	
**Family currently receives public assistance (i.e., food stamps, Medicaid)**	1100/2690 (41%)	525/1175 (45%)	0.028[2]
**Psychiatric chief complaint**	1105/2740 (40%)	542/1193 (45%)	0.003[2]
**Suicide Attempt - Lifetime**	793/2740 (29%)	379/1193 (32%)	0.075[2]

[1] Two-sided t-test with unpooled variance.
[2] Chi-squared test of no association.
SD=Standard Deviation

© 2023 Brent DA et al. *JAMA Network Open.*

eTable 3. Predicting Return Visit to ED/Hospital for Suicide Attempt/Ideation at 3-Month Follow-up (Measure and 95% CI)

Screening questionnaire	Sensitivity	Specificity	Positive Predictive Value	Negative Predictive Value
ASQ	0.922<br>(0.881-0.963)	0.587<br>(0.568-0.606)	0.126<br>(0.107-0.144)	0.991<br>(0.987-0.996)
CASSY (using cutpoint of 0.0414 where sensitivity is equal to sensitivity for ASQ)	0.922<br>(0.881-0.963)	0.620<br>(0.602-0.639)	0.135<br>(0.115-0.155)	0.992<br>(0.988-0.996)
CASSY (using cutpoint of 0.0408 where specificity is equal to specificity for ASQ)	0.940<br>(0.904-0.976)	0.587<br>(0.568-0.606)	0.128<br>(0.109-0.147)	0.993<br>(0.989-0.997)
CASSY (using optimal cutpoint of 0.0486 where sensitivity + specificity is maximized)	0.904<br>(0.859-0.949)	0.663<br>(0.645-0.681)	0.147<br>(0.126-0.169)	0.991<br>(0.986-0.995)
ED=Emergency Department; CI=Confidence Interval; ASQ=Ask Suicide-Screening Questions; CASSY=Computerized Adaptive Screen for Suicidal Youth				

© 2023 Brent DA et al. *JAMA Network Open.*

eTable 4. AUC, Sensitivity, and Specificity for Predicting Return Visit to ED/Hospital for Suicide Attempt/Ideation at 3-Month Follow-up by Screening Questionnaire Within Subgroups (Measure and 95% CI)

		AUC			Sensitivity		Specificity	
Subgroup	N	CASSY	ASQ	P-value	CASSY[1]	ASQ	CASSY[1]	ASQ
Overall	2740	0.841 (0.818-0.864)	0.754 (0.732-0.777)	<.001	0.904 (0.859-0.949)	0.922 (0.881-0.963)	0.663 (0.645-0.681)	0.587 (0.568-0.606)
Sex								
Male	991	0.874 (0.824-0.925)	0.779 (0.722-0.837)	<.001	0.838 (0.719-0.957)	0.865 (0.755-0.975)	0.777 (0.750-0.803)	0.694 (0.665-0.723)
Female	1705	0.809 (0.780-0.838)	0.729 (0.704-0.753)	<.001	0.921 (0.873-0.968)	0.937 (0.894-0.979)	0.592 (0.567-0.616)	0.521 (0.496-0.545)
Age (years)								
12-14	1339	0.853 (0.821-0.885)	0.766 (0.734-0.799)	<.001	0.894 (0.829-0.960)	0.918 (0.859-0.976)	0.691 (0.665-0.716)	0.615 (0.588-0.642)
15-17	1401	0.829 (0.796-0.862)	0.743 (0.711-0.775)	<.001	0.914 (0.852-0.975)	0.926 (0.869-0.983)	0.636 (0.610-0.662)	0.560 (0.533-0.587)
Race								
White	1618	0.822 (0.793-0.852)	0.748 (0.719-0.776)	<.001	0.909 (0.855-0.963)	0.918 (0.867-0.969)	0.635 (0.610-0.659)	0.577 (0.552-0.602)
Black or African American	469	0.849 (0.787-0.911)	0.762 (0.706-0.817)	<.001	0.852 (0.718-0.986)	0.926 (0.827-1.000)	0.686 (0.642-0.729)	0.597 (0.552-0.643)
Other/Unknown	653	0.882 (0.841-0.923)	0.767 (0.716-0.818)	<.001	0.931 (0.839-1.000)	0.931 (0.839-1.000)	0.715 (0.679-0.750)	0.603 (0.564-0.641)
Ethnicity								
Hispanic	678	0.837 (0.788-0.887)	0.772 (0.724-0.820)	0.003	0.903 (0.799-1.000)	0.935 (0.849-1.000)	0.703 (0.668-0.738)	0.609 (0.571-0.647)

© 2023 Brent DA et al. *JAMA Network Open.*

eTable 4. AUC, Sensitivity, and Specificity for Predicting Return Visit to ED/Hospital for Suicide Attempt/Ideation at 3-Month Follow-up by Screening Questionnaire Within Subgroups (Measure and 95% CI) (continued)

		AUC			Sensitivity		Specificity	
Subgroup	N	CASSY	ASQ	P-value	CASSY[1]	ASQ	CASSY[1]	ASQ
Not Hispanic	1846	0.834 (0.806-0.861)	0.743 (0.716-0.770)	<.001	0.897 (0.844-0.950)	0.913 (0.863-0.962)	0.644 (0.621-0.666)	0.573 (0.550-0.597)
Psychiatric chief complaint								
No	1635	0.873 (0.805-0.942)	0.776 (0.638-0.914)	0.158	0.545 (0.251-0.840)	0.727 (0.464-0.990)	0.889 (0.874-0.904)	0.825 (0.806-0.843)
Yes	1105	0.668 (0.626-0.710)	0.558 (0.535-0.581)	<.001	0.929 (0.889-0.969)	0.935 (0.897-0.974)	0.276 (0.247-0.304)	0.180 (0.156-0.204)

[1] Using optimal cutpoint.

AUC=Receiver Operator Area Under the Curve; ED=Emergency Department; CI=Confidence Interval; CASSY=Computerized Adaptive Screen for Suicidal Youth; ASQ=Ask Suicide-Screening Questions

© 2023 Brent DA et al. *JAMA Network Open.*

eReferences.

1. Horowitz LM, Bridge JA, Teach SJ, et al. Ask Suicide-Screening Questions (ASQ): a brief instrument for the pediatric emergency department. *Arch Pediatr Adolesc Med.* 2012;166(12):1170-1176. doi: 10.1001/archpediatrics.2012.1276

2. Posner K, Brown GK, Stanley B, et al. The Columbia–Suicide Severity Rating Scale: initial validity and internal consistency findings from three multisite studies with adolescents and adults. *Am J Psychiatry*. 2011;168(12):1266-1277. doi: 10.1176/appi.ajp.2011.10111704

3. Kroenke K, Spitzer RL, Williams JBW, Löwe B. An ultra-brief screening scale for anxiety and depression: the PHQ-4. *J Psychosom Res.* 2009;50(6):613-621. doi: 10.1176/appi.psy.50.6.613

4. Richardson LP, McCauley E, Grossman DC, et al. Evaluation of the Patient Health Questionnaire-9 Item for detecting major depression among adolescents. *Pediatrics*. 2010;126(6):1117-1123. doi:10.1542/peds.2010-0852

5. Bush K, Kivlahan DR, McDonell MB, Fihn SD, Bradley KA. The AUDIT Alcohol Consumption Questions (AUDIT-C): an effective brief screening test for problem drinking. *Arch Intern Med.* 1998;158(16):1789-1795. doi: 10.1001/archinte.158.16.1789

6. National Institute for Drug Abuse. NIDA-Modified Assist. 2013; https://www.drugabuse.gov/sites/default/files/pdf/nmassist.pdf. Accessed October 11, 2015.

7. Nock MK, Park JM, Finn CT, Deliberto TL, Dour HJ, Banaji MR. Measuring the suicidal mind: implicit cognition predicts suicidal behavior. *Psychol Sci*. 2010;21(4):511-517. doi: 10.1177/0956797610364762

© 2023 Brent DA et al. *JAMA Network Open.*

8. Martins SS, Alexandre PK. The association of ecstasy use and academic achievement among adolescents in two U.S. national surveys. *Addict Behav*. 2009;34(1):9-16. doi: 10.1016/j.addbeh.2008.07.022

9. Peleg-Oren N, Saint-Jean G, Cardenas GA, Tammara H, Pierre C. Drinking alcohol before age 13 and negative outcomes in late adolescence. *Alcohol Clin Exp Res*. 2009;33(11):1966-1972. doi: 10.1111/j.1530-0277.2009.01035.x

10. Jellinek MS, Murphy JM, Little M, Pagano ME, Comer DM, Kelleher KJ. Use of the Pediatric Symptom Checklist to screen for psychosocial problems in pediatric primary care: a national feasibility study. *Arch Pediatr Adolesc Med*. 1999;153(3):254-260. doi:10.1001/archpedi.153.3.254

11. Pagano ME, Cassidy LJ, Little M, Murphy JM, Jellinek MS. Identifying psychosocial dysfunction in school-age children: the pediatric symptom checklist as a self-report measure. *Psychol Sch*. 2000;37(2):91-106. doi:10.1002/(SICI)1520-6807(200003)37:2%3C91::AID-PITS1%3E3.0.CO;2-3

12. Centers for Disease Control and Prevention. National Youth Risk Behavior Survey – United States. YRBS Questionnaire Content - 1991-2017. 2016; https://www.cdc.gov/healthyyouth/data/yrbs/pdf/2017/YRBS_questionnaire_content_1991-2017.pdf.

13. Lloyd-Richardson EE, Perrine N, Dierker L, Kelley ML. Characteristics and functions of non-suicidal self-injury in a community sample of adolescents. *Psychol Med*. 2007;37(8):1183-1192. doi: 10.1017/S003329170700027X

© 2023 Brent DA et al. *JAMA Network Open.*

14. Klomek AB, Marrocco F, Kleinman M, Schonfeld IS, Gould M. Bullying, depression, and suicidality in adolescents. *J Am Acad Child Adolesc Psychiatry.* 2007;46:40-49. doi: 10.1097/01.chi.0000242237.84925.18

15. Klomek AB, Marrocco F, Kleinman M, Schonfeld IS, Gould MS. Peer victimization, depression, and suicidality in adolescents. *Suicide Life Threat Behav.* 2008;38(2):166-180. doi: 10.1521/suli.2008.38.2.166.

16. Sourander A, Gyllenberg D, Brunstein Klomek A, Sillanmäki L, Ilola AM, Kumpulainen K. Association of bullying behavior at 8 years of age and use of specialized services for psychiatric disorders by 29 years of age. *JAMA Psychiatry*. 2016;73(2):159-165. doi:10.1001/jamapsychiatry.2015.2419

17. Resnick MD, Bearman PS, Blum RW, et al. Protecting adolescents from harm: findings from the National Longitudinal Study on Adolescent Health. *JAMA.* 1997;278(10):823-832. doi: 10.1001/jama.278.10.823

18. Karcher MJ, Sass D. A multicultural assessment of adolescent connectedness: testing measurement invariance across gender and ethnicity. *J Couns Psychol.* 2010;57(3):274-289. doi: 10.1037/a0019357.

19. Kann L, McManus T, Harris WA, et al. Youth risk behavior surveillance - United States, 2017. *MMWR Surveill Summ*. 2018;67(8):1-114. doi:10.15585/mmwr.ss6708a1

20. Loosier PS, Dittus PJ. Group differences in risk across three domains using an expanded measure of sexual orientation. *J Prim Prev*. 2010;31(5-6):261-272. doi:10.1007/s10935-010-0228-2

21. Vrangalova Z, Savin-Williams RC. Mostly heterosexual and mostly gay/lesbian: evidence for new sexual orientation identities. *Arch Sex Behav*. 2012;41(1):85-101. doi:10.1007/s10508-012-9921-y

22. Ebesutani C, Regan J, Smith A, et al. The 10-item Positive and Negative Affect Schedule for Children, child and parent shortened versions: application of item response theory for more efficient assessment. *J Psychopathol Behav Assess.* 2012;34;191-203. doi:10.1007/s10862-011-9273-2

23. Laurent J, Catanzaro SJ, Joiner TE, Jr., et al. A measure of positive and negative affect for children: scale development and preliminary validation. *Psychol Assess.* 1999;11(3):326-338. https://doi.org/10.1037/1040-3590.11.3.326

© 2023 Brent DA et al. *JAMA Network Open.*
