# Supplementary material for: Prediction of Suicide Attempts and Suicide-Related Events Among Adolescents Seen in Emergency Departments
Source: JAMA Netw Open. 2023 Feb 15;6(2):e2255986. doi: 10.1001/jamanetworkopen.2022.55986 (PMC9932829; doi:10.1001/jamanetworkopen.2022.55986)
Supplement: Supplement 2. — Nonauthor Collaborators. Pediatric Emergency Care Applied Research Network (PECARN) Collaborators [file jamanetwopen-e2255986-s002.pdf]

Supplemental Online Content: Nonauthor Collaborators
*First name, last name, and suffix (if applicable) are required and will appear in PubMed.

*Group Name(s): Pediatric Emergency Care Applied Research Network (PECARN)							
***First Name and Middle Initial(s)**	***Last Name**	***Suffix (eg, Jr, III)**	**Academic Degrees**	**Institution**	**Location (city, state/province, country)**	**Role or Contribution, eg, chair, principal investigator**	**Group (if more than 1 Group listed in the byline) and/or Subgroup (eg, Steering Committee)**
Dale	Woolridge		MD	University of Arizona	Tucson, AZ, USA	Principal Investigator	
Marie	Kay		BA	(PECARN) Data Coordinating Center at the University of Utah	Salt Lake City, UT, USA	Project Manager	
Michelle	Robinson		BS	(PECARN) Data Coordinating Center at the University of Utah	Salt Lake City, UT, USA	Lead Project Manager	
Casey	Evans		BS	(PECARN) Data Coordinating Center at the University of Utah	Salt Lake City, UT, USA	Clinical Data Manager	
Renee	Kuhn		BS, CCRP	(PECARN) Data Coordinating Center at the University of Utah	Salt Lake City, UT, USA	Project Manager	
Esther	Ullman		MSW	Survey Research Operations at the University of Michigan	Ann Arbor, MI, USA	Project Lead	
Lisa	Carn		BA	Survey Research Operations at the University of Michigan	Ann Arbor, MI, USA	Production Manager	
Maureen	O'Brien		MSW	Survey Research Operations at the University of Michigan	Ann Arbor, MI, USA	Project Lead, previously Sr. Project Manager	
Lisa	Colpe		PhD, MPh	Formerly of the National Institute of Mental Health	Bethesda, MD	ED-STARS Steering Committee Member	
Anthony	Spirito		PhD	Warren Alpert Medical School at Brown University	Providence, RI, USA	Scientific Advisor Board Member	
Erin	Ryan		MPH	Warren Alpert Medical School at Brown University	Providence, RI, USA	Study Coordinator	
Thomas	Chun		MD	Warren Alpert Medical School at Brown University	Providence, RI, USA	Principal Investigator	
Afton	Kirk-Johnson		PhD	Formerly at UPMC Western Psychiatric Hospital<br>Now at the Department of Pediatrics University of Pittsburgh	Pittsburgh, PA, USA	Editorial Support	
