# Supplementary material for: Prediction of Suicide Attempts and Suicide-Related Events Among Adolescents Seen in Emergency Departments
Source: JAMA Netw Open. 2023 Feb 15;6(2):e2255986. doi: 10.1001/jamanetworkopen.2022.55986 (PMC9932829; doi:10.1001/jamanetworkopen.2022.55986)
Supplement: Supplement 3. — Data Sharing Statement [file jamanetwopen-e2255986-s003.pdf]

Data Sharing Statement

Brent. Prediction of Suicide Attempts and Suicide-Related Events Among Adolescents Seen in Emergency Departments. *JAMA Netw Open*. Published February 15, 2023.
doi:10.1001/jamanetworkopen.2022.55986

Data
Data available: Yes
Data types: Deidentified participant data, Data dictionary
How to access data: https://nda.nih.gov/
When available: With publication

Supporting Documents
Document types: None

Additional Information
Who can access the data: Anyone requesting the data
Types of analyses: Individual approaches the data repository with data requests explaining the purpose of their request.
Mechanisms of data availability: Without investigator support
